# Hydrophobic mismatch demonstrated for membranolytic peptides, and their use as molecular rulers to measure bilayer thickness in native cells

**DOI:** 10.1038/srep09388

**Published:** 2015-03-25

**Authors:** Ariadna Grau-Campistany, Erik Strandberg, Parvesh Wadhwani, Johannes Reichert, Jochen Bürck, Francesc Rabanal, Anne S. Ulrich

**Affiliations:** 1 University of Barcelona, Faculty of Chemistry, Department of Organic Chemistry, Martí i Franquès, 1, 08028, Barcelona, Spain; 2 Karlsruhe Institute of Technology (KIT), Institute of Biological Interfaces (IBG-2), POB 3640, 76021 Karlsruhe, Germany; 3 KIT, Institute of Organic Chemistry, Fritz-Haber-Weg 6, 76131 Karlsruhe, Germany

## Abstract

Hydrophobic mismatch is a well-recognized principle in the interaction of transmembrane proteins with lipid bilayers. This concept was extended here to amphipathic membranolytic α-helices. Nine peptides with lengths between 14 and 28 amino acids were designed from repeated KIAGKIA motifs, and their helical nature was confirmed by circular dichroism spectroscopy. Biological assays for antimicrobial activity and hemolysis, as well as fluorescence vesicle leakage and solid-state NMR spectroscopy, were used to correlate peptide length with membranolytic activity. These data show that the formation of transmembrane pores is only possible under the condition of hydrophobic matching: the peptides have to be long enough to span the hydrophobic bilayer core to be able to induce vesicle leakage, kill bacteria, and cause hemolysis. By correlating the threshold lengths for biological activity with the biophysical results on model vesicles, the peptides could be utilized as molecular rulers to measure the membrane thickness in different cells.

Membrane-active antimicrobial peptides (AMPs) are found in almost all types of organisms and constitute a host defense system against microorganisms^1^. Over 2000 AMPs are known^2^ and can be classified according to origin, activity or structure^1^,^2^,^3^. Linear cationic amphipathic α-helices are the most common AMPs and also have the widest antimicrobial activity spectrum, some well-known examples being magainins from frogs^4^ and LL-37 from humans^5^. Besides exploring natural AMPs from a wide variety of organisms, much effort has also been spent to obtain new peptides with improved activities. One approach is to modify natural sequences, while another strategy is based on the design of amphipathic sequences from scratch, as in the case of MSI-103 with the regular repeat (KIAGKIA)_3_-NH_2_^3^. This heptameric motif was based on the sequence of PGLa, a member of the magainin family of antimicrobial peptides from the African frog *Xenopus laevis*^6^. The full-length peptide was optimized by simplifying the sequence to contain only four types of amino acid, and by increasing the positive charge while maintaining the overall amphipathic character^3^. MSI-103 was found to have a higher antimicrobial activity than the parent peptide, while hemolytic side effects were reduced^3^,^7^.

The parent peptide of MSI-103, PGLa, as well as magainin 2 from the same family, have been proposed to permeabilize membranes by forming pores across the lipid bilayer^8^,^9^,^10^. Pores are attributed to peptides in a transmembrane orientation, forming either a so-called “barrel-stave” as described for alamethicin^11^, or a “toroidal wormhole” that includes lipid head groups as reported, e.g., for melittin^12^. For melittin, there is strong indication of membranous pores from X-ray studies, but the orientation of the peptide is not visible from these experiments^12^. Complementary methods, such as solid-state NMR and oriented circular dichroism, have been used to monitor directly the alignment of helical peptides and thereby discriminate a surface-bound state from a stable transmembrane alignment and from any tilted state in between^13^,^14^,^15^,^16^,^17^,^18^,^19^. This way, MSI-103 was shown to self-assemble and start to insert into lipid bilayers in a similar manner to its parent peptide PGLa, but at a lower threshold concentration^7^,^15^,^20^,^21^,^22^,^23^,^24^.

The same kind of solid-state NMR studies have also been instrumental in deciphering the phenomenon of hydrophobic matching, which is known to affect the tilt angle and assembly of fully inserted transmembrane protein segments^25^,^26^,^27^,^28^,^29^. It is generally recognized that long membrane-spanning helices can become tilted to match the bilayer thickness^25^,^30^,^31^,^32^, and lipid bilayers are in turn able to adjust their thickness or lipid phase to accommodate very short hydrophobic segments^30^,^32^. So far, the concept of hydrophobic mismatch has only been applied to fully hydrophobic transmembrane helices.

The effect of peptide length on the activity of amphipathic AMPs has been investigated in numerous studies^33^,^34^,^35^,^36^,^37^,^38^,^39^,^40^. However, we are not aware of any attempt to monitor or interpret their membranolytic activity in terms of the matching between peptide length and membrane thickness. Therefore, to extend the elegant principle of hydrophobic matching for the first time to amphipathic helices, we investigate here a series of MSI-derived peptides with different lengths. Given the underlying repeat unit KIAGKIA, we call them KIA[n] peptides, where n is the number of amino acids in each peptide. Our original aim was twofold: (i) to find the optimal length of peptides with high antimicrobial activity and low hemolytic side effects, and (ii) to re-examine the classical pore formation hypothesis, which has been under some critical debate recently^41^,^42^,^43^,^44^,^45^. Namely, if the amphipathic peptides were to form stable or transient pores built from genuinely transmembrane helices, they would clearly require a minimum length to span the membrane. In that case one would expect to find a threshold minimum length for activity, which would also have to vary with the thickness of the target membrane. On the other hand, it has been argued that this class of antimicrobial peptides can kill bacteria either in a detergent-like fashion by permeabilizing the lipid bilayer according to the “carpet model”, where the peptides remain crowded on the membrane surface^46^, or by translocating the membrane to find some intracellular target^47^. In those cases, even peptides that are much too short to span the membrane could be active^48^,^49^ (unless of course the translocation process itself occurs via a transient transmembrane pore). To study the fundamental effect of membrane thickness on peptide activity in complementary biological and biophysical ways, we used antibacterial and hemolysis assays on living cells, as well as fluorescence leakage experiments with model lipid vesicles composed of synthetic lipids with defined acyl chain length. Additionally, solid-state NMR was employed to examine the orientation of peptides in DMPC (1,2-dimyristoyl-*sn*-glycero-3-phosphatidylcholine) bilayers, in order to find out whether a length-dependent re-alignment of the amphipathic helices occurs in this well-defined membrane system.

## Results

### Peptide synthesis and characterization by CD

A total of nine peptides of the KIA series with lengths 14–28 amino acids were synthesized as listed in Table 1, with a non-perturbing ^15^N-NMR label at the backbone amide of Ala-10. Circular dichroism (CD) was used to compare their secondary structure in different media. In phosphate buffer, all peptides showed random coil spectra (see Supplementary Figure S1A). In small unilamellar DMPC/DMPG (1,2-dimyristoyl-*sn*-glycero-3-phosphatidylglycerol) (3:1 mol/mol) vesicles, at a peptide-to-lipid molar ratio (P/L) of 1:50, the line shapes were all very similar to one another, typical of α-helices (see Supplementary Figure S1B). Clearly, all peptides are unstructured in solution but fold as α-helices when bound to membranes. A deconvolution of the CD spectra was performed, giving 68–83% α-helical content of the peptides, as shown in Table 1. A more complete table of the deconvolution result is shown in Table S1.

### Antimicrobial activity

The effect of the peptides on bacteria was determined using a minimal inhibition concentration (MIC) assay. As seen in Table 2 (and in Supplementary Table S2), there is a clear correlation between peptide length and biological activity, as a minimum length is required for peptides to be antimicrobially effective. Interestingly, this threshold length differs for the different bacterial strains. In *Escherichia coli* (DSM 1103), KIA14 and KIA15 are completely inactive, while KIA17 and KIA19 show some activity, and KIA21 and longer peptides show high activity. In *Pseudomonas aeruginosa* (DSM 1117), KIA19 and shorter analogs are inactive, KIA21 and KIA22 somewhat active, while for full activity 24 or more amino acids are needed. In *Staphylococcus aureus* (DSM 1104), the threshold is more distinct: KIA19 and shorter peptide are inactive, whereas KIA21 and longer peptides are highly active. In *Enterococcus faecalis* (DSM 2570), hardly any activity is found for KIA22 and shorter peptides, while KIA24 and KIA26 show some medium activity (compared to the shorter peptides), and only KIA28 is much more active than the shorter peptides. In summary, there is a clear threshold length in all bacterial strains, as peptides shorter than the threshold have much lower activity than the longer ones. We also note that the activity of the control peptide PGLa (with 21 amino acids) most closely resembles that of KIA17 and KIA19.

### Hemolysis

**Figure 1:**
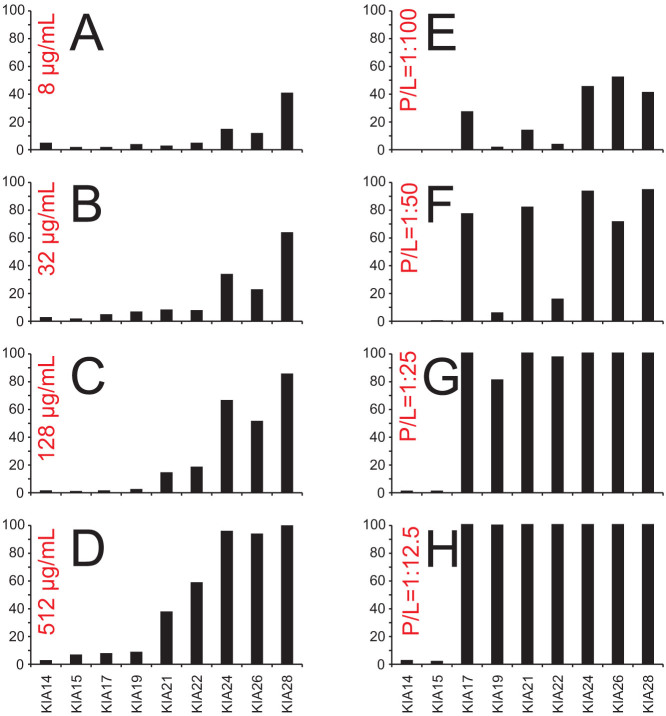
(A–D) Hemolysis of KIA peptides, measured at four different peptide concentrations. (E–H) Leakage induced by KIA peptides in POPC/POPG (1:1) vesicles, measured at four different peptide-to-lipid ratios (P/L). At high peptide concentration the short peptides give no leakage, but KIA17 and longer peptides give 100% leakage.

Amphipathic antimicrobial peptides show membranolytic effects not only against bacteria, but they can also permeabilize eukaryotic cells such as erythrocytes. Hemolytic activities of the KIA peptides are summarized in Figure 1A–D (and in Supplementary Table S3), recorded for several different peptide concentrations. A clear jump in activity is observed for the series of peptides with different lengths, as already seen in the MIC assays. The short KIA14 to KIA19 gave very low hemolysis, even at a high peptide concentration of 512 μg/mL. KIA21 and KIA22 showed considerable activity only at or above 128 μg/mL, while KIA24 and longer peptides caused strong hemolysis already at 8 μg/mL and reach 100% hemolysis at the highest concentration used. In summary, the threshold length of KIA peptides to permeabilize erythrocyte membranes is around 21 to 22 amino acids, and superimposed on this jump is a monotonous increase in hemolysis with peptide length and concentration.

### Vesicle leakage

In the MIC and hemolysis assays above with living cells, it was not possible to control the composition of the membrane lipids, and the acyl chain lengths are not known. Therefore, we performed complementary *in vitro* experiments, by measuring the leakage of fluorescent dye from small unilamellar vesicles with well-defined membrane thickness. Different synthetic lipids were chosen with distinctly different acyl chain lengths. In all cases, a 1:1 (mol/mol) mixture of zwitterionic phosphatidylcholine (PC) and anionic phosphatidylglycerol (PG) head groups was used. Anionic lipids are known to be the main components of bacterial membranes, which contain in many cases well over 50% PG^50^. Furthermore, a negative charge is necessary to attract the water-soluble cationic peptides electrostatically to the vesicles^48^,^51^. Leakage curves were measured at different P/L ratios, over 10 minutes after addition of the vesicle suspension to the peptide solution. At very low peptide concentration only partial leakage could be achieved, but with a higher P/L of 1:25 or more, there was in almost all cases either no leakage (≤10%) or almost complete leakage (≥85%), as illustrated in Figure 1E–H for POPC/POPG (1-palmitoyl-2-oleoyl-*sn*-glycero-3-phosphatidylcholine/1-palmitoyl-2-oleoyl-*sn*-glycero-3-phosphatidylglycerol). A clear length dependent effect is thus observed, indicating that leakage can only occur when the peptides are long enough to span the hydrophobic part of the lipid bilayer. The results for all lipid systems under investigation are summarized for P/L = 1:12.5 in Table 3.

In the typical membrane lipids POPC/POPG (with C16:0/C18:1 chains) no leakage was observed for KIA14 and KIA15, but KIA17 and longer peptides induced almost complete leakage. In thick bilayers composed of DErPC/DErPG (1,2-dierucoyl-*sn*-glycero-3-phosphatidylcholine/1,2-dierucoyl-*sn*-glycero-3-phosphatidylglycerol) (with di-C22:1 chains) there was also a threshold, but now only KIA24 and longer peptides gave rise to leakage. We also examined a set of lipid mixtures with zwitterionic PC and anionic PG, whose acyl chain lengths were significantly mismatched to one another. Since lateral phase segregation is expected to occur in these cases, we intended to find out whether either the anionic or the uncharged lipid domains would be targeted by the peptide. The data in Table 3 show that the threshold length of the peptides is dominated by the charged PG component. The pattern in the well-matched POPC/POPG mixture is identical to that in the length-unbalanced DMoPC/DOPG (1,2-dimyristoleoyl-*sn*-glycero-3-phosphatidylcholine, di-C14:1-PC/1,2-dioleoyl-*sn*-glycero-3-phosphatidylglycerol, di-18:1-PG) and DErPC/POPG systems: KIA17 or longer peptides gave total leakage, even if the PC lipids were significantly shorter or longer than the anionic component. Less extreme is the case for POPC/DErPG, where 21 residues are required to induce leakage, hence this threshold is in between that of POPC/POPG and DErPC/DErPG.

An interesting fluctuating pattern emerges not only in Table 3, but also in the leakage and hemolysis panels of Figure 1, where it had been left uncommented so far. Certain peptides are obviously less active than their next shorter neighbor (for example, KIA19 is less active than KIA17 in DErPC/POPG, and KIA26 is less active than KIA24 in DErPC/DErPG). In all these cases, membrane perturbation is seen to occur also for the longer peptide, but to a lower extent or with a lower rate (see kinetic data in Supplementary Figure S2). The common denominator of the more active peptides is the fact that they all carry a hydrophobic unit Ile-Ala at the C-terminus, while the longer, less active peptides all end with a charged Lys.

### Solid-state NMR

**Figure 2:**
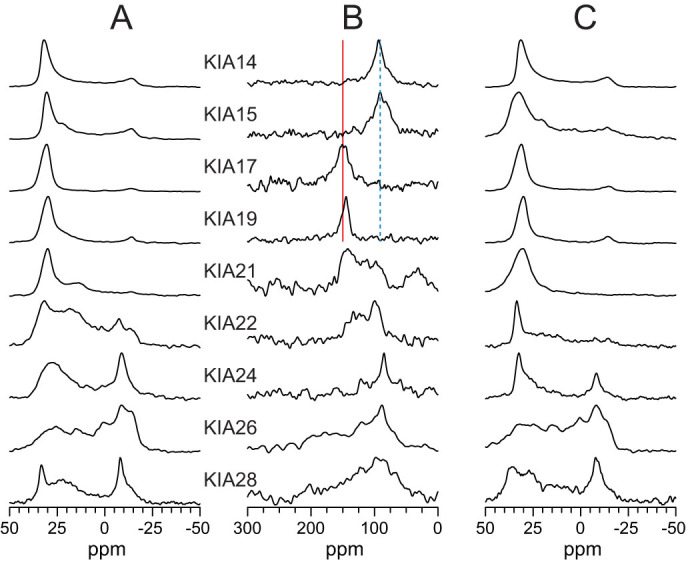
Solid-state NMR spectra of KIA peptides in DMPC at P/L = 1:20. (A) ^31^P-NMR spectra before ^15^N-NMR. (B) ^15^N-NMR spectra. (C) ^31^P-NMR spectra after ^15^N-NMR. The ^15^N-NMR signal of KIA14 and KIA15 at 90 ppm (blue dotted line) is typical of the surface-bound state. It shifts to 150 ppm (red line), indicating an inserted state for KIA17 and some longer peptides. For KIA22 and the even longer peptides, broad powder line shapes are seen in ^15^N-NMR (with a peak also at 90 ppm), and the ^31^P-NMR spectra show a perturbed lipid bilayer.

Pore formation induced by an amphipathic peptide must be accompanied by its insertion into a transmembrane state. The actual orientation of an α-helical peptide in a bilayer can be determined from a solid-state NMR analysis in oriented membrane samples, by observing the chemical shift of a ^15^N-label in the peptide backbone^52^. All KIA peptides were synthesized with a ^15^N-amide at Ala-10 to perform such ^15^N-NMR experiments in a well-defined lipid bilayer system. DMPC is the most convenient lipid that aligns well in oriented NMR samples and does not suffer from oxidation^13^,^14^,^20^,^53^,^54^,^55^. ^15^N-NMR spectra of the different KIA peptides reconstituted in DMPC at a P/L ratio of 1:20 are given in Figure 2B, and Figure 2A/2C shows the corresponding ^31^P-NMR line shapes of the phospholipid matrix. The ^31^P-NMR spectra were collected before and after the ^15^N-experiments, in order to check for any perturbations in the lipid bilayer that may be induced by the peptide, or that could occur as a result of sample dehydration during the long ^15^N-measurements.

A striking length-dependent re-alignment behavior of the KIA peptides is observed by solid-state NMR. The ^15^N-NMR signals of KIA14 and KIA15 at 90 ppm are indicative of a surface-bound orientation, while KIA17 and KIA19 give a chemical shift around 150 ppm representing a highly tilted state, in which the peptides reach deeply into the membrane. This fits with the leakage results in POPC/POPG where KIA14 and KIA15 were not active, while KIA17 and longer peptides induced leakage (see Figure 1). KIA21 shows a broader line shape with ^15^N-signals between 150 ppm and 90 ppm, suggesting that this peptide is no longer oriented in a uniform way but instead assumes a range of different tilt angles. The longest peptides from KIA22, KIA24, KIA26 to KIA28 then become completely disordered, as their spectra turn into broad powder patterns (giving signals between 220 and 60 ppm, with a maximum around 90 ppm). Interestingly, these long peptides are also found to induce considerable perturbation in the membrane, as seen in the ^31^P-NMR spectra (broad upfield powder component with a maximum around 0 ppm in panels 2A/2C). These ^31^P and ^15^N powder line shapes indicate that the longest peptides no longer have a preferred alignment and strongly disturb the lipid bilayer (at least for the high P/L ratio used here to obtain sufficient sensitivity for the ^15^N-NMR measurements). The NMR data in DMPC can thus be interpreted in terms of a length-dependent insertion of the KIA peptides; the shortest ones are surface-bound and remain inactive, but when the peptide length matches that of the lipid bilayer the helices can insert deeply into the membrane and become active. If the peptides are too long to span the hydrophobic bilayer core, they will no longer take on a well-defined alignment but lead to considerable disorder in the lipid matrix, which is also a sign of membrane damage, which can occur in several ways.

## Discussion

In this study, we have systematically explored how the length of amphipathic α-helical peptides affects their membranolytic activity. Nine model peptides with lengths from 14 to 28 amino acids were constructed from simple repeats of the sequence KIAGKIA. CD showed that all peptides are mostly α-helical in DMPC/DMPG vesicles (Figure S1, Table 1). We have previously performed solid-state ^19^F-NMR experiments in both DMPC/DMPG and in bacterial and erythrocyte membranes of several AMPs, and found the same structure and orientation of peptides in both DMPC/DMPG and in biological membranes^56^,^57^, supporting the conclusion that the KIA peptides are α-helical also in biological membranes.

We found that (i) their antimicrobial activities exhibit a distinct length-dependent threshold, which interestingly differs for different bacterial strains. Similarly, (ii) the hemolytic effect increases abruptly with peptide length, and (iii) a fluorescence leakage assay with synthetic lipid vesicles also showed that a minimum peptide length is required that matches the bilayer thickness.

**Figure 3:**
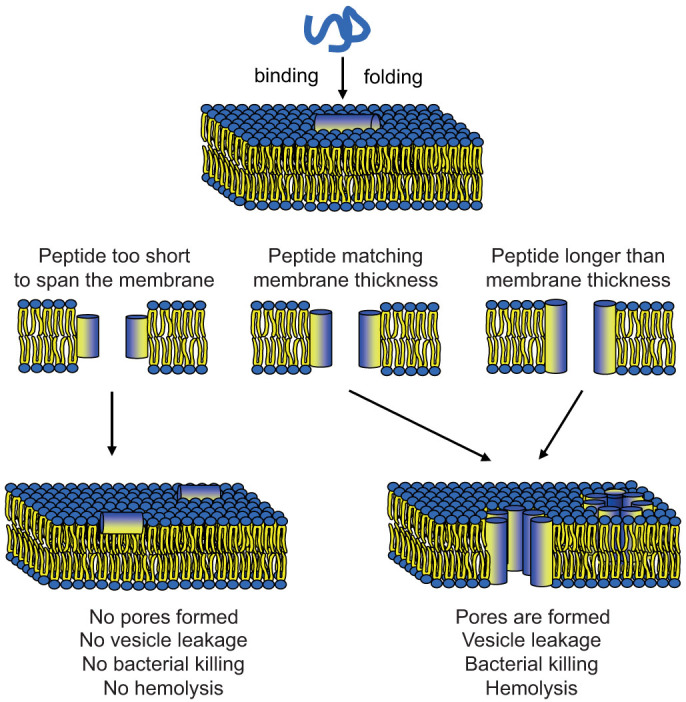
Schematic illustration of the pore formation mechanism proposed to occur for KIA peptides. The peptides are unstructured in solution but form amphipathic α-helices when bound to the membrane, according to CD spectroscopy. If the peptide is too short to span the hydrophobic lipid bilayer core, no pores are formed and the helix remains flat on the membrane surface. If the peptide length matches the bilayer thickness, a transmembrane pore can form with peptides in an inserted, upright orientation. (Pores are depicted as barrel-staves for simplicity of drawing, though they may well be of the toroidal wormhole type with lipid head groups lining the pore).

All these results are consistent with the classical - but recently questioned^41^,^42^,^43^,^44^,^45^ - hypothesis of peptide pore formation. The intuitive idea is that amphipathic peptides form transmembrane pores in the lipid bilayer with essentially upright helices, according to the Shai-Matsuzaki-Huang model of a “toroidal wormhole”^58^,^59^ or a “barrel-stave” pore^60^. The measured leakage can be explained by assuming that only a minor part of the membrane-bound peptides are involved in forming the pores^61^. Figure 3 illustrates that such pores should only be feasible if the peptide is long enough to span the membrane, while shorter peptides should stay on the membrane surface and remain inactive. Our results do not only provide new and unambiguous evidence to support this concept, which had so far been based merely on indirect observations. Furthermore, the systematic leakage data now allow us to perform a quantitative analysis of the length-dependent effects, as the lipid acyl chains are well defined and the membrane thickness of the vesicles is known.

The length of an ideal α-helical peptide is 1.5. Å per residue. The residues close to the termini might not be in an ideal α-helical configuration, which could make the peptide longer or shorter depending on the exact local structure, which might also fluctuate over time. We will estimate the length of the KIA peptides to be as a first approximation 1.5 Å per residue, but this might be a few Å away from the real value. The hydrophobic thickness has been determined experimentally for many synthetic lipid bilayers. For lipids where no data is available, the thickness can be approximated using formulae according to Marsh^62^. Table 3 thus gives the peptide lengths and hydrophobic thickness of the lipid bilayers used, indicating at the same time whether or not a certain peptide/lipid combination leads to vesicle leakage. For DErPC/DErPG there is a very clear correlation, as only those peptides longer than the hydrophobic thickness of 34.4 Å^63^ can induce leakage. In POPC/POPG, the KIA17 peptide with an approximate length of 25.5 Å already induces leakage, being only marginally shorter than the bilayer thickness of about 27.1 Å^63^,^64^. It should be noted that the hydrophobic thickness is not the full membrane thickness, but only the thickness of the hydrophobic core. For example, in POPC, the hydrophobic thickness is 27.1 Å, while the distance between the average planes of the phosphate groups on each side of the bilayer is 37.6 Å, and the “steric thickness” between the outer surfaces of the lipid head groups is 45.1 Å^63^. In other words, the amphipathic helices only need to be long enough to span the hydrophobic part of the membrane to be active, not the entire membrane. If the peptides are longer, they can also easily span part of the more polar head group region and become more tilted.

This structural behavior is nicely seen in the solid-state ^15^N- and ^31^P-NMR analyses of the different KIA peptides, carried out in a well-defined model membrane system, as previously explained in more detail^21^,^24^. A distinct length-dependent re-alignment is observed here in DMPC, as illustrated in Figure 3. When the helices are too short they remain surface-bound and inactive (i.e. KIA14 and KIA15). Only KIA17 with a threshold length of 25.5 Å is found to insert in a well-defined manner, being perfectly matched to the hydrophobic thickness of 25.4 Å^65^ and obviously able to form a stable membrane-spanning pore. When the peptides are too long (i.e. 31.5 Å for KIA21) they become disordered and lead to considerable membrane perturbation, which still accounts for their high membranolytic activity.

From these findings we conclude that only peptides long enough to span the hydrophobic membrane core can form pores that are responsible for leakage. Of course, there are still numerous situations in which leakage is weak or does not occur at all, even though the peptide is in principle long enough. Many reasons can be named for such lack in activity. A straightforward explanation holds in those cases where the total peptide concentration is simply too low, as seen for example in the hemolysis and leakage panels of Figure 1A/B/E. The total weight of peptide in these assays always makes an underlying contribution to the observed activity, but cannot account for the abrupt length-dependent jumps discussed here. Another scenario with unexpectedly low activity has been noted for the KIA peptides terminating with a charged residue. In all assays (MIC, hemolysis, leakage) those peptides with a hydrophobic Ile-Ala at the C-terminus tend to be more active than their next longer neighbor with Lys. These subtle effects seem to be due to the actual residues at the end of the helix, as Lys renders the C-terminus more polar and seems to make its insertion into the bilayer core less favorable. We have indeed shown in a recent comprehensive solid-state NMR structure analysis of KIA21 (formerly called MSI-103, [KIAGKIA]_3_-NH_2_) that it is the hydrophobic C-terminus that starts to tilt into the membrane, in line with the fact that the sequence carries a charged Lys at the N-terminus like all KIA peptides^20^,^24^. This kinetic effect is supported by the slower rates of leakage observed for those peptides with a C-terminal Lys (see Supplementary Figure S2).

Another reason for a low membranolytic activity of AMPs can often be attributed to a lack of initial membrane binding due to electrostatic attraction. The bacterial membranes employed above in the MIC tests are rich in anionic lipids anyway, and also in the hemolysis assays the KIA peptides showed a sufficient membrane binding affinity. To encourage membrane binding, we included anionic lipids in the fluorescence vesicle leakage assays (though this was not necessary in the solid-state NMR samples as they contain no excess water into which the peptide could escape from the oriented multibilayers). In the mixed vesicle preparations where the acyl chain lengths of the PC and PG lipids were not the same, we found that the threshold length for the peptides is determined largely by the anionic lipid component. DMoPC/DOPG and DErPC/POPG show the same threshold as POPC/POPG, even though the PC lipids have very different acyl chain lengths [19.2 Å for DMoPC with di-14:1 chains^62^; 34.4 Å for DErPC with di-22:1 chains^63^], while DOPG has a length (27.5 Å)^64^ comparable to that of POPG (27.8 Å)^64^. For the case of POPC/DErPG, the threshold lies in between that of POPC/POPG and DErPC/DErPG. These observations lead to the conclusion that the cationic KIA peptides are attracted to the membrane surface by the negatively charged PG lipids, with which they will most likely form PG-rich domains, as previously shown for KIA21 (MSI-103)^66^. Even if all these conditions are fulfilled (electrostatic attraction, sufficiently high peptide concentration, suitable termini), the peptide must nevertheless be long enough to span this lipid domain in order to form pores.

Synthetic lipids with known acyl chain lengths were employed in the leakage experiments, but natural biomembranes contain a variety of lipids with different chains and head groups, plus other components like membrane proteins and cholesterol. Therefore, the actual membrane hydrophobic thickness is unknown in our biological assays on bacteria and erythrocytes. Assuming that the cells are killed by pores, formed the same way as in the vesicle leakage experiments, we can now use the KIA peptides as molecular rulers to obtain information about their effective membrane thickness. Both in Gram-positive and Gram-negative bacteria, the relevant membrane is the (inner) cytoplasmic membrane.

In *E. coli*, we found that the shortest peptide able to kill the bacteria is KIA17, which is at the same time the shortest peptide able to induce leakage in POPC/POPG membranes. Thus, the hydrophobic membrane thickness of *E. coli* should be similar to POPC/POPG, i.e. around 27 Å, at least for the anionic lipid components or domains thereof. According to an X-ray scattering analysis, the observed thickness of the *E. coli* inner membrane, measured as the average distance between phosphate groups on opposite sides of the bilayer, is 37.5 Å^67^. This value fits very well with the corresponding distance measured in POPC bilayer, which was found to be 37.6 Å, and translates into a hydrophobic thickness of 27.1 Å^63^.

For other bacteria we have not found any data on membrane thickness in the literature, but we can now use our results on KIA peptides to estimate them. *E. faecalis* was killed only by KIA24 and longer peptides, which were also needed to induce leakage in DErPC/DErPG. Therefore, the hydrophobic thickness of the *E. faecalis* membrane should be similar to DErPC/DErPG, namely around 34 Å. In *P. aeruginosa* and *S. aureus* the membranes should be around 31 Å thick, as these bacteria are killed by KIA21, but not by any of the shorter peptides. For hemolysis of erythrocytes, the threshold was not so clear-cut, but only KIA21 and longer peptides showed a pronounced effect at high concentration, so these eukaryotic membranes seem to have a similar hydrophobic thickness as those of *S. aureus* and *P. aeruginosa*, namely around 31 Å. In one study, from electron microscopy the hydrophobic thickness of erythrocyte membranes was estimated to be ~25 Å, and from X-ray diffraction a head group separation of ~48 Å was found^68^, which considering experimental uncertainties is compatible with our results here.

In the literature, many groups have studied the effect of peptide length on the activity of AMPs^33^,^34^,^35^,^36^,^37^,^38^,^39^,^40^, but to the best of our knowledge no quantitative studies on the matching of peptide length with membrane thickness have been reported. For magainin 2 with 23 amino acids, it was found that the full activity against *E. coli* was retained when 3 N-terminal residues were removed; with 4 residues removed the activity was reduced, and when 5 or 6 amino acids were removed the activity was completely lost^33^. These results imply that 19 amino acids are needed for proper activity against *E. coli*, similar to what we find here for the KIA peptides. Since magainin 2 is believed to form pores in a similar way to PGLa^8^,^9^,^10^, the parent peptide in the design of the KIA peptides, this similar threshold length further supports the pore-forming hypothesis.

In several previous reports, amphipathic α-helical model peptides had been designed and their antimicrobial activities were tested as a function of length. In some cases, like in the present study, a minimum length was found to be necessary for activity, and all longer analogs were also active^34^,^35^,^36^. We can thus assume that these kinds of peptides kill bacteria in the same manner as the KIA peptides, by forming pores across the membrane with peptides in a transmembrane orientation. However, in some other studies a different behavior was found, with a maximum activity for a certain peptide length, and less activity for both longer and shorter sequences^37^,^38^,^39^,^40^. Often, the highest activity was found for peptides too short to be expected to form pores, namely for around 15^37^,^38^ or even 12 amino acids^39^,^40^. Also some other short amphipathic α-helical peptides, like BP100 with 11 amino acids^48^,^49^, have a high antimicrobial activity, but seem much too short to form transmembrane pores. For those peptides, a different mechanism must be responsible for their membranolytic activity, for example the “carpet” model^46^.

## Conclusions

Using a series of model amphipathic α-helical peptides of different length from 14 to 28 amino acids, we have been able to demonstrate that (i) antimicrobial peptides can align in the membrane to form transmembrane pores, (ii) they require a distinct bilayer length dependent threshold to be active, (iii) this length dependence can be explained by hydrophobic (mis)matching (a well-known fundamental concept that had so far only been developed for and applied to hydrophobic peptides or protein segments^25^,^30^,^31^,^32^ and now is extended also to amphipathic helices, and (iv) our biological results show that the length-dependent activity can be used to estimate the thickness of different bacterial (or eukaryotic) cellular membranes *in vivo*. We also expect this study to be of help for the design of new AMPs that could be useful as future antibiotic drugs.

## Methods

### Materials

Peptide synthesis reagents and Fmoc-protected amino acids were purchased from Merck Biosciences (Darmstadt, Germany) and/or Iris Biotech (Marktretwitz, Germany). ^15^N-labelled amino acids were purchased from Cambridge Isotope Laboratories (Andover, MA, USA) and were Fmoc-protected using Fmoc-Cl as described previously^69^. Solvents for peptide synthesis were purchased from Merck (Darmstadt, Germany) or from Biosolve (Valkenswaard, Netherlands) and solvents for HPLC purification were obtained from Fischer Scientific (Geel, Belgium). The lipids 1,2-dierucoyl-*sn*-glycero-3-phosphatidylcholine (DErPC), 1,2-dierucoyl-*sn*-glycero-3-phosphatidylglycerol (DErPG), 1,2-dimyristoyl-*sn*-glycero-3-phosphatidylcholine (DMPC), 1,2-dimyristoyl-*sn*-glycero-3-phosphatidylglycerol (DMPC), 1,2-dimyristoleoyl-*sn*-glycero-3-phosphatidylcholine (DMoPC), 1,2-dioleoyl-*sn*-glycero-3-phosphatidylglycerol (DOPG), 1-palmitoyl-2-oleoyl-*sn*-glycero-3-phosphatidylcholine (POPC), 1-palmitoyl-2-oleoyl-*sn*-glycero-3-phosphatidylglycerol (POPG), and 1,2-dioleoyl-*sn*-glycero-3-phosphoethanolamine-N-(lissamine rhodamine B sulfonyl) (Rhod-PE) were obtained from Avanti Polar Lipids (Alabaster, USA). The fluorescent probes 8-amino-naphtalene-1,3,6-trisulfonic acid sodium salt (ANTS) and pxylenebis(pyridinium)bromide (DPX) were obtained from Invitrogen - Molecular Probes (Karlsruhe, Germany).

### Peptide synthesis

KIA peptides were synthesized on an automated Syro II multiple peptide synthesizer (MultiSynTech, Witten, Germany), using standard Fmoc solid phase peptide synthesis protocols^20^,^70^. The crude peptides were purified using a high-pressure liquid chromatography (HPLC) device from JASCO (Groß-Umstadt, Germany) on a preparative Vydac C18 column using a water/acetonitrile gradient supplemented with 5 mM HCl. The purified peptides were characterized by using analytical LC (Agilent; Waldbron, Germany) coupled to an ESI mass spectrometer (μTOF Bruker, Bremen, Germany) and were found to be over 95% pure.

### Circular dichroism spectroscopy (CD)

CD samples were prepared by co-solubilizing DMPC and DMPG (3:1 mol/mol) in chloroform/methanol 3:1 (v/v). After drying, the lipid film was dispersed in phosphate buffer (10 mM, pH 7) and homogenized by vortexing. Small unilamellar vesicles (SUVs) for CD samples were generated by sonication in a high-power ultrasonic bath with a beaker-shaped sonotrode (UTR 200, Hielscher, Germany). CD spectra were recorded on a J-815 spectropolarimeter (JASCO, Groß-Umstadt, Germany) between 260 and 185 nm at 0.1-nm intervals, using 1-mm quartz glass cells (Suprasil; Hellma, Müllheim, Germany) at 20°C, as reported previously^51^. The peptides were measured at 20°C in 10 mM sodium phosphate buffer (pH 7.0) and at 30°C in lipid vesicles composed of DMPC/DMPG (3:1). Typical peptide concentrations of the final samples in phosphate buffer was 36–72 μM, and for the vesicle samples 30 μM with a peptide-to-lipid molar ratio (P/L) of 1:50. An averaged baseline of the pure solvent or lipid matrix, respectively, was subtracted. Finally, the spectra were converted to mean residue ellipticities (MRE) by using the weighed-in peptide amount and the volume of the sample for concentration determination. The peptide stocks contain some salt after HPLC, and some water might be present, but the material had to be weighed in, as the KIA peptides do not contain any aromatic amino acids for calibration by UV/VIS absorption. Although this ambiguity in peptide concentration leads most probably to an underestimation of the magnitude of the MRE values we attempted a quantitative deconvolution of CD data to estimate the relative contents of different secondary structure elements. If part of the weighted material was not peptide, this leads to an underestimating of the MRE, which also leads to an underestimation of the helical content^71^. The values obtained are thus the minimum helical content of each peptide.

Secondary structure estimation from CD spectra was performed using the CONTIN-LL program, which is based on the ridge regression algorithm^72^,^73^. The algorithm is provided by the DICHROWEB on-line server^74^. The quality of the fit between experimental and back-calculated spectrum corresponding to the estimated secondary structure fractions was assessed from the normalized root mean square deviation (NRMSD), with a value <0.1 considered as a good fit^74^.

### MIC (minimum inhibitory concentration) assay

Antimicrobial activity was measured by a standard MIC assay, carried out with Gram-positive *Staphylococcus aureus* (DSM 1104) and *Enterococcus faecalis* (DSM 2570) and with Gram-negative *Escherichia coli* (DSM 1103) and *Pseudomonas aeruginosa* (DSM 1117), as previously reported^53^. Briefly, bacteria were grown in Müller-Hinton medium at 37°C overnight. Microtiter plates (96 wells of 100 μL) were filled with 50 μL of Müller-Hinton medium, and serial 2-fold dilutions of peptides were arranged in quadruple. The two final rows of each plate remained without peptide, so that the penultimate data point served as the positive control (no peptide), and the final one as the negative control (not inoculated). 50 μL of bacterial suspension (OD = 0.2) was added to the wells (except for the final row of each plate) to give a final concentration of 10^6^ colony-forming units (CFU)/mL. The plates were incubated at 37°C for 20 h, and the MIC value was determined visually on the basis of turbidity as the lowest peptide concentration inhibiting bacterial growth.

### Hemolysis assay

Hemolytic activity was examined with a serial 2-fold dilution assay as previously described^7^. Citrate phosphate dextrose-stabilized blood bags with erythrocyte suspensions of healthy donors were obtained from the blood bank of the local municipal hospital (Städtisches Klinikum, Karlsruhe, Germany). The erythrocytes, previously washed, were incubated with peptide solutions at 37°C for 30 min with gentle shacking. The tubes were centrifuged at 13000 rpm for 10 min to pellet the cells, and the absorbance at 540 nm was recorded against a negative control (cells without peptide, accounting for autohemolysis). The percentage of lysis was then calculated relative to 100% lysis induced by 1% Triton X-100. The absorbance measurements were repeated three times, and the averaged values were used.

### Vesicle leakage assay

For the leakage experiments^75^, the buffer in which the vesicles were prepared contained the fluorophor ANTS (12.5 mM), the quencher DPX (45 mM), 50 mM NaCl, and 10 mM HEPES (pH 7.5). Liposomes were prepared by co-dissolving PC/PG 1:1 (mol/mol) mixtures of the lipids with different chain length in CHCl_3_/MeOH (3:1 v/v), together with 10^−2^ mol% Rhod-PE by which the lipid loss during vesicle preparation (extrusion and gel filtration, see below) could be quantified. The peptide-to-lipid ratio (P/L) is given in mol/mol. The lipid mixture was dried under N_2_(g) and left to dry under vacuum overnight. The obtained thin film was then re-suspended in the buffer which contained the fluorophor and the quencher by vigorous vortexing, followed by 10 freeze-thaw cycles. Large unilamellar vesicles (LUV) were obtained by 21-fold extrusion (Avanti Mini Extruder; Avanti Polar Lipids, Alabaster, AL) of the liposomes through a Nuclepore polycarbonate membrane (pore size 100 nm, Whatman - GE Healthcare Europe, Freiburg, Germany) at a temperature 20°C above the lipid phase transition. Unencapsulated dye was removed by gel filtration using spin columns filled with Sephacryl 100-HR (Sigma-Aldrich, Taufkirchen, Germany), and equilibrated with an elution buffer (150 mM NaCl, 10 mM HEPES, pH 7.5) which balances the internal vesicle osmolarity.

Leakage of entrapped ANTS was monitored by fluorescence dequenching of ANTS^76^. Fluorescence measurements were performed in a thermostatted cuvette with constant stirring at 30°C in the same buffer as used for gel filtration on a FluoroMax2 spectrofluorimeter (HORIBA Jobin Yvon, Unterhaching, Germany) by setting the ANTS emission to 510 nm (5 nm slit) and its excitation to 355 nm (5 nm slit). The vesicles (100 μM lipid final concentration) of the desired composition were added to the cuvette containing the peptide at the P/L ratio to be tested (from 1 mM stock solutions in water). The level of 0% leakage corresponded to the fluorescence of the vesicles immediately after their addition, while 100% leakage was the fluorescence value obtained after addition of a 0.25 vol-% Triton X-100 after 10 min.

### Solid-state NMR

Macroscopically oriented NMR samples were prepared by co-dissolving appropriate amounts of peptides and lipids (in 300 μl methanol, 100 μl CHCl_3_, and 10–20 μl milliQ-water), and spreading onto 23 thin glass plates of dimensions 9 mm × 7.5 mm × 0.08 mm (Marienfeld Laboratory Glassware, Lauda-Königshofen, Germany). The peptide-to-lipid ratio (P/L) is given in mol/mol. The plates were dried in air for 1 h, followed by drying under vacuum overnight. They were stacked and placed into a hydration chamber with 96% relative humidity at 48°C for 18–24 h, before wrapping the stack in parafilm and plastic foil for the NMR measurements.

All solid-state NMR measurements were carried out on a Bruker Avance 500 or 600 MHz spectrometer (Bruker Biospin, Karlsruhe, Germany) at 308 K. ^31^P-NMR was used to check the quality of the lipid orientation in the samples, using a Hahn echo sequence with ^1^H decoupling and phase cycling. ^1^H-^15^N cross polarization experiments using a CP-MOIST pulse sequence^77^ were performed using a double-tuned probe with a low-E flat-coil resonator (3 mm × 9 mm cross section), employing a ^1^H and ^15^N radiofrequency field strength of 65 kHz during the cross polarization, and 36 kHz ^1^H SPINAL16 decoupling during acquisition. A mixing time of 500 μs was used, and 10000 to 30000 scans were accumulated. The acquisition time was 10 ms and the recycle time 4 s. The ^15^N chemical shift was referenced using the signal of an ammonium sulfate sample set to 26.8 ppm. The oriented membrane samples were placed in the flat-coil probe such that the lipid bilayer normal was aligned parallel to the magnetic field.

## Supplementary Material

Supplementary Information Supplementary information

## Figures and Tables

**Table 1:** Synthesized peptides used in this study. The highlighted Ala-10 was labelled with ^15^N at the backbone amide

Peptide	Sequence	Helicity^a^/%	Length^b^/Å
KIA14	KIAGKIA KI**A**GKIA-NH_2_	74	21
KIA15	KIAGKIA KI**A**GKIA K-NH_2_	69	22.5
KIA17	KIAGKIA KI**A**GKIA KIA-NH_2_	83	25.5
KIA19	KIAGKIA KI**A**GKIA KIAGK-NH_2_	82	28.5
KIA21^c^	KIAGKIA KI**A**GKIA KIAGKIA-NH_2_	83	31.5
KIA22	KIAGKIA KI**A**GKIA KIAGKIA K-NH_2_	68	33
KIA24	KIAGKIA KI**A**GKIA KIAGKIA KIA-NH_2_	73	36
KIA26	KIAGKIA KI**A**GKIA KIAGKIA KIAGK-NH_2_	71	39
KIA28^d^	KIAGKIA KI**A**GKIA KIAGKIA KIAGKIA-NH_2_	76	42

^a^From CD data deconvolution using the CONTIN-LL algorithm^74^.

^b^Approximate length, assuming fully helical peptides with a length of 1.5 Å per residue for an ideal α-helix.

^c^Also called MSI-103^3^.

^d^Also called MSI-1127^3^.

**Table 2:** MIC values (μg/mL) for KIA peptides in four different bacterial strains. Inactive peptides are marked in bold for each strain

Peptide	Gram-negative		Gram-positive	
	*E. coli*	*P. aeruginosa*	*S. aureus*	*E. faecalis*
KIA14	**>256**	**>256**	**>256**	**>1024**
KIA15	**>256**	**>256**	**>256**	**>1024**
KIA17	32	**256**	**256**	**>1024**
KIA19	32	**256**	**>256**	**>1024**
KIA21	4	64	8	**1024**
KIA22	4	32	16	**1024**
KIA24	4	16	4	64
KIA26	4	16	8	64
KIA28	8	16	8	16
PGLa (control)	32	**256**	64	**>1024**

**Table 3:** Leakage induced by KIA peptides in vesicles with different acyl chain composition. The percentage of leakage is taken after 10 minutes at P/L = 1:12.5, relative to the level upon the addition of Triton X-100 (defined as 100% leakage). The length of the peptides and the hydrophobic thickness of the bilayers (corresponding to the pure lipids) are also given. Inactive peptides are marked in bold for the different lipid systems

	Lipid	DMoPC/DOPG	POPC/POPG	DErPC/POPG	POPC/DErPG	DErPC/DErPG
Peptide	Length (Å)	19.2^a^/27.5^b^	27.1^c^/27.8^b^	34.4^c^/27.8^b^	27.1^c^/34.4^c^	34.4^c^/34.4^c^
KIA14	21	**8**	**3**	**5**	**2**	**1**
KIA15	22.5	**3**	**2**	**3**	**2**	**0**
KIA17	25.5	100	100	84	**17**	**1**
KIA19	28.5	100	100	30	**4**	**2**
KIA21	31.5	100	100	98	82	**4**
KIA22	33	100	100	85	26	**6**
KIA24	36	100	100	100	100	89
KIA26	39	100	100	100	97	10
KIA28	42	100	100	100	100	84

^a^Value from Ref. 62.

^b^Value from Ref. 64.

^c^Value from Ref. 63.
