# Supplementary material for: Hydrophobic mismatch demonstrated for membranolytic peptides, and their use as molecular rulers to measure bilayer thickness in native cells
Source: Sci Rep. 2015 Mar 25;5:9388. doi: 10.1038/srep09388 (PMC5224518; doi:10.1038/srep09388)
Supplement: Supplementary Information [file srep09388-s1.doc]

**Supplementary information**

**Hydrophobic mismatch demonstrated for membranolytic peptides, and their use as molecular rulers to measure bilayer thickness in native cells**

Ariadna Grau-Campistany^1^, Erik Strandberg^2^, Parvesh Wadhwani^2^, Johannes Reichert^2^, Jochen Bürck^2^, Francesc Rabanal^1^ & Anne S. Ulrich^2,3^

^1^ University of Barcelona, Faculty of Chemistry, Department of Organic Chemistry, Martí i Franquès, 1, 08028, Barcelona, Spain; ^2^ Karlsruhe Institute of Technology (KIT), Institute of Biological Interfaces (IBG-2), POB 3640, 76021 Karlsruhe, Germany; ^3^ KIT, Institute of Organic Chemistry, Fritz-Haber-Weg 6, 76131 Karlsruhe, Germany.

Correspondence and requests for materials should be addressed to A.S.U. (email: anne.ulrich@kit.edu).

**Supplementary Table S1.** Secondary structure fractions of KIA peptides in DMPC/DMPG (3:1) vesicles, evaluated from the CD spectra using the CONTIN-LL algorithm.

| **Peptide** | **Percentage of secondary structure element** | | | | |  |
| --- | --- | --- | --- | --- | --- | --- |
|  | **α-helix** | **β-sheet** | **β-turn** | **unordered** | **total** | **NRMSD^a^** |
| KIA14 | 74.1 | 3.4 | 5.2 | 17.3 | 100.0 | 0.042 |
| KIA15 | 68.7 | 5.2 | 7.8 | 18.3 | 100.0 | 0.050 |
| KIA17 | 82.7 | 3.2 | 3.0 | 11.2 | 100.1 | 0.041 |
| KIA19 | 81.7 | 3.0 | 3.1 | 12.2 | 100.0 | 0.038 |
| KIA21 | 83.0 | 3.2 | 2.8 | 11.0 | 100.0 | 0.031 |
| KIA22 | 67.7 | 4.2 | 6.6 | 21.5 | 100.0 | 0.029 |
| KIA24 | 72.6 | 3.5 | 6.6 | 17.3 | 100.0 | 0.026 |
| KIA26 | 71.1 | 4.7 | 7.1 | 17.2 | 100.1 | 0.026 |
| KIA28 | 76.2 | 4.0 | 5.2 | 14.7 | 100.1 | 0.022 |

^a^NRMSD: normalized root mean square deviation between the experimental and back-calculated CD spectrum. A value < 0.1 is considered as a good fit.

Supplementary Table S2. MIC values (µM) for KIA peptides in four different bacterial strains. Inactive peptides are marked in grey for each strain.

| **Peptide** | **Gram negative** | | **Gram positive** | |
| --- | --- | --- | --- | --- |
|  | *E. coli* | *P. aeruginosa* | *S. aureus* | *E. faecalis* |
| KIA14 | >164 | >164 | >164 | >657 |
| KIA15 | >149 | >149 | >149 | >595 |
| KIA17 | 17 | 134 | 134 | >537 |
| KIA19 | 15 | 120 | >120 | >481 |
| KIA21 | 1.7 | 28 | 3.5 | 443 |
| KIA22 | 1.6 | 13 | 6.5 | 414 |
| KIA24 | 1.5 | 6.0 | 1.5 | 24 |
| KIA26 | 1.4 | 5.6 | 2.8 | 22 |
| KIA28 | 2.6 | 5.2 | 2.6 | 5.2 |
| PGLa (control) | 15 | 119 | 30 | >477 |

**Supplementary Table S3.** Hemolytic activity of KIA peptides at different peptide concentrations, given both as μg/mL and as μM. Peptides showing small effects even at the highest tested concentration are marked in grey.

|  | **Peptide concentration** | | | | | | | |
| --- | --- | --- | --- | --- | --- | --- | --- | --- |
|  | ***8 μg/mL*** | | ***32 μg/mL*** | | ***128 μg/mL*** | | ***512 μg/mL*** | |
| **Peptide** | **Hemolysis**  **(%)** | ***µM*** | **Hemolysis**  **(%)** | ***µM*** | **Hemolysis**  **(%)** | ***µM*** | **Hemolysis**  **(%)** | ***µM*** |
| KIA14 | 5 | *5.1* | 3 | *20.5* | 2 | *82.1* | 3 | *328.4* |
| KIA15 | 2 | *4.6* | 2 | *18.6* | 1 | *74.3* | 7 | *297.3* |
| KIA17 | 2 | *4.2* | 2 | *16.8* | 2 | *67.1* | 8 | *268.5* |
| KIA19 | 4 | *3.8* | 5 | *15.0* | 3 | *60.2* | 9 | *240.7* |
| KIA21 | 3 | *3.5* | 7 | *13.8* | 15 | *55.4* | 38 | *221.5* |
| KIA22 | 5 | *3.2* | 8 | *12.9* | 19 | *51.7* | 59 | *206.9* |
| KIA24 | 15 | *3.0* | 34 | *12.0* | 67 | *48.1* | 96 | *192.6* |
| KIA26 | 12 | *2.8* | 23 | *11.1* | 52 | *44.4* | 94 | *177.8* |
| KIA28 | 41 | *2.6* | 64 | *10.4* | 86 | *41.8* | 100 | *167.1* |

**Supplementary Figures**

**Figure S1.** Circular dichroism spectra of KIA peptides, (A) in 10 mM phosphate buffer with a peptide concentration of 36-72 µM (~0.1 mg/mL); (B) in the presence of DMPC/DMPG (3:1) small unilamellar vesicles at P/L=1:50 and with peptide concentrations of 30 µM (~0.04-0.08 mg/mL).

**Figure S2.** Time course of leakage induced by KIA peptides in POPC/POPG vesicles at P/L=1:50. At time zero the vesicle solutions were added to the buffer solutions which contained the peptides, and after 600 s Triton-X100 was added to achieve complete (100%) lysis of the vesicles. KIA14 and KIA15 induce no leakage (< 2%). KIA17 and longer peptides provoke leakage, but each with very different rates. Two groups can be discerned, and in each group the rate increases with peptide length. However, KIA17, KIA21, KIA24 and KIA28 show always fast kinetics, and they carry the hydrophobic unit Ile-Ala at their C-terminus. KIA19, KIA22 and KIA26 terminate with Lys and show slower kinetics relative to their neighbors in the first group.
